# MEC-12/alpha tubulin regulates mitochondrial distribution and mitophagy during oxidative stress in *C. elegans*

**DOI:** 10.17912/micropub.biology.001232

**Published:** 2024-06-24

**Authors:** Fivos Borbolis, Myrsini Kteniadaki, Konstantinos Palikaras

**Affiliations:** 1 Department of Physiology, School of Medicine, National and Kapodistrian University of Athens, Athens, Greece; 2 Athens International Master's Programme in Neurosciences, Department of Biology, National and Kapodistrian University of Athens, Athens, Greece

## Abstract

Mitophagy, the selective removal of dysfunctional mitochondria, is pivotal for the maintenance of neuronal function and survival. MEC-12/α-tubulin contributes to neuronal physiology through the regulation of microtubule assembly, intracellular transport and mitochondrial distribution. However, its role in mitochondrial dynamics and mitophagy remains obscure. Here, we demonstrate that MEC-12 influences mitochondrial morphology under basal conditions and regulates the axonal mitochondrial population. Impairment of MEC-12 results in compromised axonal mitophagy under both basal conditions and oxidative stress. Our results uncover the critical role of MEC-12/α-tubulin for maintaining a healthy mitochondrial population in axons and highlight the complex interplay between microtubules, mitophagy and neuronal health.

**Figure 1. MEC-12/alpha tubulin regulates mitochondrial morphology and mitophagy under basal conditions and oxidative stress:**
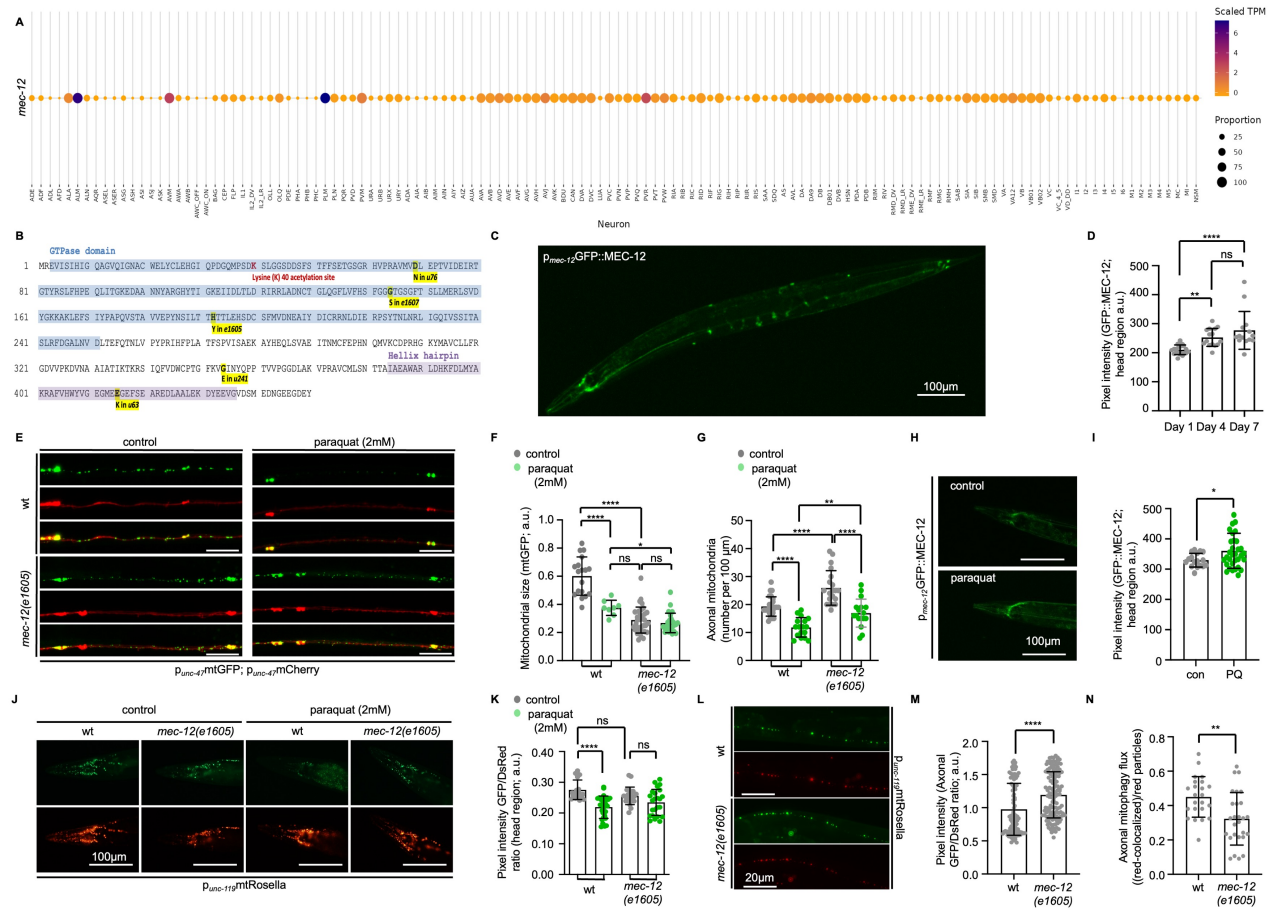
( **A** ) Single-cell RNA sequencing data confirming the pan-neuronal expression pattern of the *mec-12* gene (data retrieved from https://www.cengen.org). ( **B** ) The amino acid sequence of MEC-12 with indicated mutations: acetylated lysine residue (red), D69N ( *u76* ), G144S ( *e1607* ), H192Y ( *e1605* ), G354E ( *u241* ), E425K ( *u63* ).( **C** ) Transgenic animals expressing the fluorescent protein GFP fused with MEC-12 under the endogenous *mec-12* promoter. The GFP::MEC-12 protein displays neuronal localization. Scale bar, 100μm. ( **D** ) Quantitative analysis of GFP::MEC-12 accumulation in neurons with age (ns *P* >0.05; ** *P* <0.001; **** *P* <0.0001; One-way ANOVA). ( **E** ) Representative confocal microscopy images of wild type (wt) and *mec-12(e1605)* mutants co-expressing mitochondria-targeted GFP and cytosolic mCherry in GABAergic motor neurons under control conditions and after exposure to 2mM paraquat. Scale bars, 20μm ( **F** ) Measurement of mitochondrial size in wt and *mec-12(e1605)* mutants, under control and paraquat-induced oxidative stress conditions (ns *P* >0.05; * *P* <0.01; **** *P* <0.0001; One-way ANOVA). ( **G** ) Effects of paraquat treatment on axonal mitochondrial number in wt and *mec-12(e1605)* mutants (** *P* <0.001; **** *P* <0.0001; One-way ANOVA). ( **H** ) Increase in GFP::MEC-12 levels upon paraquat treatment, indicating stress-induced up-regulation (head region; Scale bars, 100μm). ( **I** ) Quantitative analysis of GFP::MEC-12 protein levels in response to paraquat treatment (* *P* <0.01; unpaired *t* -test). ( **J** ) Representative fluorescent images of wt and *mec-12(e1605)* transgenic nematodes expressing mtRosella under the pan-neuronal promoter *unc-119* treated with paraquat. Scale bars, 100μm. ( **K** ) MEC-12 is required for mitophagy induction (reduced GFP/DsRed ratio) upon paraquat treatment (ns *P* >0.05; **** *P* <0.0001; One-way ANOVA). ( **L** ) Representative fluorescent images of axonal mtRosella signals were obtained using wt and *mec-12(e1605)* transgenic nematodes, which express mtRosella pan-neuronally. Scale bars, 20μm. ( **M** ) The increased mtRosella ratio of *mec-12(e1605)* mutants suggests diminished basal mitophagy in axons (**** *P* <0.0001; unpaired *t* -test). ( **N** ) Mitophagic flux is reduced in the axons of *mec-12(e1605)* mutant nematodes (** *P* <0.001; unpaired *t* -test).

## Description

Neurons depend on a fine-tuned interplay between mitochondrial biogenesis, mitochondrial dynamics and mitochondrial selective autophagy (known as mitophagy) to sustain their energy homeostasis and support their functions (Borbolis & Palikaras, 2022; Collier et al., 2023; Misgeld & Schwarz, 2017; Trigo et al., 2022) . Microtubules, key components of the neuronal cytoskeleton, ensure precise mitochondrial transport and positioning along axons, dendrites and synapses, thereby promoting efficient energy distribution and calcium buffering across extensive neuronal processes (Cason & Holzbaur, 2022; Cheng & Sheng, 2021; Kapitein & Hoogenraad, 2015; Zaninello & Bean, 2023) . Impaired microtubule dynamics can lead to altered mitochondrial transport and activity, which in turn could compromise neuronal connectivity, function and viability, potentially contributing to the development of neurodegenerative diseases (Cason & Holzbaur, 2022; Cheng & Sheng, 2021; Zaninello & Bean, 2023) . Therefore, understanding the interplay between microtubules and mitochondrial quality control is essential for unraveling the mechanisms of neuronal resilience and pathology.

MEC-12 is the homolog of mammalian alpha-tubulin in *C. elegans* and plays a pivotal role in the structural and functional integrity of neuronal microtubules (Bounoutas et al., 2009; Fukushige et al., 1999; Zheng et al., 2017) . Emerging findings suggest that MEC-12 mediates the assembly and stabilization of microtubules supporting neurite growth and neuronal morphology (Bounoutas et al., 2009; Zheng et al., 2017) . MEC-12 displays a pan-neuronal expression pattern that is particularly pronounced in mechanosensory neurons, where its deficiency directly impacts touch sensitivity by modulating the mechanotransduction process ( **Figure 1A** ; Bounoutas et al., 2009; Fukushige et al., 1999; Zheng et al., 2017). Mutations in MEC-12 could affect the distribution of mechanoreceptor channel complexes and selectively impair touch sensitivity and disrupt microtubule assembly. Furthermore, MEC-12 mutations affect mechanoreceptor currents, suggesting a specialized role for MEC-12-modulated microtubules in mechanotransduction, distinct from their general functions in the maintenance of neuronal structure and protein transport (Bounoutas et al., 2009; Fukushige et al., 1999; Zheng et al., 2017) .

Although all the MEC-12 mutations result in touch sensitivity defects, different mutant alleles display a variety of phenotypes affecting microtubule formation and structure, intracellular transport, protein distribution, and acetylation levels. Among the available *mec-12* mutants, *mec-12 ( e1605 )* and *mec-12 ( u63 )* are touch insensitive but still exhibit normal microtubules formation (Bounoutas et al., 2009; Chalfie & Au, 1989; Fukushige et al., 1999; Zheng et al., 2017) . Notably, *mec-12 ( e1605 )* and *mec-12 ( u63 )* nematodes carry point mutations in the microtubule-associated proteins (MAP) binding /GTPase domain and the N-terminal H12-helix of α-tubulin, respectively ( **Figure 1B** ). A recent study utilized *mec-12 ( u63 )* expressing mitochondria-targeted GFP in mechanosensory neurons and identified altered mitochondrial distribution in PLM neurons, with fewer mitochondria in the anterior axonal region and more in the posterior, compared to wild type animals (Teoh et al., 2022) . These results suggest a novel role for MEC-12 in the modulation of mitochondrial homeostasis within neurons, underlining its broader impact on cellular health and response to physiological stress.

We found that MEC-12 displays a pan-neuronal expression pattern, confirmed by recent single-cell RNA sequencing analysis ( **Figure 1A, C ) (** www.cengen.org ) (Fukushige et al., 1999; Solinger et al., 2010) . We assessed the localization and expression levels of MEC-12 using transgenic *C. elegans* strains expressing MEC-12 fused with the GFP protein at its N-terminus, driven by the endogenous *mec-12* promoter ( **Figure 1C** ). Additionally, we monitored GFP:: MEC-12 protein levels and found that MEC-12 is increased in neurons with age ( **Figure 1D** ). These results suggest that age-dependent accumulation of GFP:: MEC-12 might correspond to impaired microtubule dynamics, potentially compromising neuronal integrity and function, and eventually affecting intracellular transport.

We then examined mitochondrial morphology and distribution in wild type and *mec-12 ( e1605 )* mutants using transgenic animals co-expressing mitochondria-targeted GFP, known to be localized in the outer mitochondrial membrane, and cytosolic mCherry in GABAergic motor neurons. Both GFP:: MEC-12 expressing nematodes and single-cell RNA sequencing analysis confirmed the expression of MEC-12 in GABAergic motor neurons ( **Figure 1A, C** ). In *mec-12 ( e1605 )* mutants, we observed an increased number of smaller mitochondria within axonal compartments ( **Figure 1 E, F** ). Oxidative stress induced by paraquat treatment leads to a reduction in mitochondrial number of GABAergic motor neurons (Zaninello et al., 2020) . Then, we examined whether MEC-12 is required for mitochondrial distribution upon paraquat exposure. *mec-12 ( e1605 )* nematodes displayed increased mitochondrial number compared to their wild type counterparts, whereas mitochondrial size remained unchanged under oxidative stress ( **Figure 1E -G** ). Notably, GFP:: MEC-12 levels increased in response to paraquat treatment indicating the critical role of MEC-12 in the regulation of mitochondrial distribution and morphology during stress conditions ( **Figure 1H, I** ).

Mitophagy is stimulated in response to challenging conditions to eliminate damaged organelles and preserve cellular physiology (Borbolis & Palikaras, 2022; Palikaras et al., 2018; Picca et al., 2023) . Treatment with paraquat triggers oxidative stress and has been shown to induce mitophagy in *C. elegans* (Palikaras et al., 2015; Zaninello et al., 2020) . We used paraquat to induce mitochondrial dysfunction and analyzed the subsequent induction of mitophagy using the mitochondria-targeted Rosella biosensor (Cummins et al., 2019; Fang et al., 2019; Palikaras et al., 2015) . Rosella is a purpose-built reporter comprising of a fast-maturing pH-insensitive DsRed fused to a pH-sensitive GFP variant (Rosado et al., 2008; Palikaras et al., 2019) . Thus, mitophagy levels can be signified by monitoring the ratio of GFP to DsRed intensity. Quantitative analysis in the head region of *C. elegans* , which includes the nerve ring and multiple neuronal cell bodies, uncovered that neuronal mitophagy is induced following paraquat exposure in wild type animals. In contrast, *mec-12 ( e1605 )* mutants displayed a significant mitophagy impairment ( **Figure 1J, K** ). Small and globular mitochondria are prerequisites for mitophagy initiation (Burman et al., 2017; Kageyama et al., 2014) . *mec-12 ( e1605 )* mutants exhibited more circular and smaller mitochondria within axonal processes, thus we investigated the levels of axonal mitophagy under non-stressed conditions. Notably, *mec-12 ( e1605 )* mutants displayed an elevated mtRosella (GFP/DsRed) ratio and decreased mitophagic flux in axons, suggesting defective basal mitophagy ( **Figure 1L -N** ).

The altered mitochondrial dynamics and impaired stress response in *mec-12* mutants underscore the critical role of MEC-12 /α-tubulin in the maintenance of mitochondrial integrity through mitophagy. Presumably, the H192Y substitution presented in *mec-12 ( e1605 )* nematodes disrupts the appropriate interaction between microtubules and mitochondria that might regulate organellar positioning and transport of axonal organelles to the cell bodies of GABAergic neurons for degradation. In agreement with this notion, the existence of a more fragmented mitochondrial pool that consists of a higher number of smaller organelles in the axons of *mec-12 ( e1605 )* animals suggests that mitophagy can be locally initiated and induce organelle fission in such distal parts of these neuronal cells but cannot be completed due to the impairment of mitochondrial transport. This disruption could contribute to the accumulation of dysfunctional mitochondria in axonal processes, exacerbating cellular stress and potentially accelerating neurodegeneration. Future studies should investigate the differential effects of additional *mec-12* mutant alleles ( **Figure 1B** ) and/or perform rescue experiments. Therefore, further experiments are warranted to uncover the full range of physiological consequences and mechanistic details related to mitochondrial distribution, as well as both basal and stress-induced mitophagy. Moreover, the unique cellular architecture of neurons should be considered to further examine any potential differences among neuronal cell populations. These efforts could enhance the therapeutic potential of modulating microtubule-associated proteins to enhance mitochondrial function and resilience in neurodegenerative conditions.

## Methods

*C. elegans* maintenance

We followed standard procedures for *C. elegans* strain maintenance (Stiernagle, 2006) . Nematode rearing temperature was kept at 20 °C.

Paraquat treatment

2- or 4-day-old adult hermaphrodites were transferred to plates containing 2 mM paraquat (methyl viologen dochliride, Sigma Aldrich) seeded with *E. coli* OP50 bacteria. Animals were imaged after 24 hours at 20 ^o^ C.

Microscopy and quantification

Nematodes were immobilized in a 20 mM tetramisole/M9 buffer drop on microscopic slides, sealed with coverslips and analyzed with the EVOS M7000 system (Thermo-fisher Scientific) or Zeiss LSM 900 confocal microscope. Quantification of the mean pixel intensity or particle analyses were performed by using the Fiji software. Mitophagic flux in mtRosella worms was assessed by using the the Fiji software **.** The mitophagy flux is calculated by the equation (number of red mitochondria – number of (red + green) mitochondria / number of red mitochondria. This equation reflects the total number of mitochondria minus the number that are still intact (indicated by the presence of GFP signal colocalized with the red signal), divided by the total number of mitochondria.

Statistical analysis

Statistical analysis was performed using GraphPad Prism 9. Data are reported as the mean values ± standard deviation (SD), unless otherwise stated. For statistical analyses, P values were calculated by unpaired Student's t-test and one-way ANOVA with Tukey's multiple comparisons test.

## Reagents

**Table:** 

**Strain**	**Genotype**	**Available from**
CB3284	*mec-12 ( e1605 )III *	CGC
GU864	*ngIs9* [p *_mec-12_* GFP:: MEC-12 ]III; p *_ttx-3_* RFP *; lin-15(+) *	Solinger et al., 2010
EG6531	*oxIs608* [p *_unc-47_* mCherry]; *oxEx1182* [p *_unc-47_* TOMM-20 ::GFP]	E.M. Jorgensen lab; Rawnson et al., 2014
KPA126	*mec-12 ( e1605 )* ; *oxIs608* [p *_unc-47_* mCherry]; *oxEx1182* [p *_unc-47_* TOMM-20 ::GFP]	Palikaras lab
IR1864	N2; *Ex001* [p *_unc-119_* TOMM-20 ::Rosella; pRF4]	Palikaras lab
